# Lowest nocturnal systolic blood pressure is related to heavy proteinuria and outcomes in elderly patients with chronic kidney disease

**DOI:** 10.1038/s41598-021-85071-2

**Published:** 2021-03-12

**Authors:** Xinru Guo, Shuang Liang, Wenling Wang, Ying Zheng, Chun Zhang, Xiangmei Chen, Guangyan Cai

**Affiliations:** 1 Medical School of Chinese PLA, Department of Nephrology, The First Medical Centre, Chinese PLA General Hospital, Beijing, China; 2 Department of Nephrology, The First Medical Centre, Chinese PLA General Hospital, 28 Fuxing Road, Haidian District, Beijing, China; 3 Department of Nephrology, The Fifth Medical Center of Chinese PLA General Hospital, Beijing, China; 4 Department of Nephrology, Xinjiang Armed Police Crops Hospital, Xinjiang Uygur Autonomous Region, Xinjiang, China

**Keywords:** Nephrology, Risk factors

## Abstract

Ambulatory blood pressure monitoring (ABPM) can produce many variables, of which the lowest nocturnal systolic blood pressure (LNSBP) currently used in calculating morning surge is occasionally overlooked in recent kidney studies compared with other ABPM parameters. We explored the clinical effects of LNSBP in elderly patients with chronic kidney disease (CKD) in a multicenter, observational cohort study. A total of 356 elderly patients with CKD from 19 clinics were included in this analysis. We used multiple logistic regression and survival analyses to assess the associations between the lowest nocturnal systolic blood pressure and heavy proteinuria and kidney disease outcomes, respectively. The median age was 66 years, and 66.6% were men. The median eGFR was 49.2 ml/min/1.73 m^2^. Multivariate logistic regression analysis demonstrated that LNSBP (OR 1.24; 95% CI 1.10–1.39; P < 0.001; per 10 mmHg) was associated with heavy proteinuria. During the median follow-up of 23 months, 70 patients (19.7%) had a composite outcome; of these, 25 initiated dialysis, 25 had 40% eGFR loss, and 20 died. Cox analysis showed that the renal risk of LNSBP for CKD outcomes remained significant even after adjusting for background factors, including age, sex, medical history of hypertension and diabetes, smoking status, eGFR, 24-h proteinuria, and etiology of CKD (HR 1.18; 95% CI 1.06–1.32; P = 0.002; per 10 mmHg). Concentrating on LNSBP could be valuable in guiding antihypertensive treatment to control heavy proteinuria and improve renal prognosis in elderly CKD patients.

## Introduction

Chronic kidney disease (CKD) can arise from heterogeneous diseases affecting 11–16% of the population worldwide^1^, in which diabetes mellitus and hypertension are the top two causes of CKD in the majority of counties^2^. It is well established that elevated blood pressure (BP) strongly indicates the progression of renal function and mortality in CKD or non-CKD populations^3–5^. In the past few decades, 24-h ambulatory blood pressure monitoring (ABPM) has exerted a better impact on evaluating the status of blood pressure throughout the day, distinguishing mask or white-coat hypertension in CKD patients more effectively than office blood pressure^6–8^. Hypertension management guidelines consistently have suggested ABPM as a better method to diagnose hypertension and can identify the proper time of antihypertensive drug administration^9,10^. ABPM also has a prognostic effect on the incidence and progression of renal damage^9,11^.

Heavy proteinuria is one of the essential diagnostic factors for nephrotic syndrome, which is commonly recognized as greater than 3.5 g measured by a timed 24-h urine collection method^12^. Clinical and experimental data have indicated that heavy proteinuria is associated with the formation of renal interstitial fibrosis and contributes to the progression of renal failure^13^. The NHANES study showed that proteinuria was an independent risk factor for hypertension and independently associated with blood pressure (BP) control in CKD^14^. BP control reduces the incidence of albuminuria^15^. However, the association between 24-h proteinuria and 24-h ambulatory blood pressure in elderly CKD patients remains uncertain because of their cumbersome, time-consuming procedures, and expenditure in clinical practice. ABPM generates an abundance of data. Researchers have evaluated BP at different times, such as morning, daytime, and nighttime blood pressure. However, the lowest nocturnal systolic blood pressure (LNSBP), which is currently used to calculate the morning surge, is scarcely studied in CKD patients. We were particularly interested in the LNSBP whether it has an association with heavy proteinuria and can predict CKD outcomes, which has not been specifically addressed to the best of our knowledge.

## Results

### Baseline characteristic of the analyzed population

Baseline characteristics are shown in Table 1. A total of 356 patients (median age 66 years, 66.6% males) from 19 clinics were included in this analysis with a median eGFR of 49.2 ml/min per 1.73 m^2^. A great part of them (73.9%) had hypertension, and greater than one-third of the population had diabetes. As presented in Table 1, no differences were noted between the LNSBP tertiles in age, smoking status, BMI, serum lipids, eGFR or etiology of CKD. ABPM parameters were all increased in patients from the highest LNSBP tertile with the exception of 24-h heart rate. We also found a trend toward a higher percentage of males, hypertension, diabetes, massive proteinuria, calcium channel blocker users, and anemia in the higher LNSBP tertile.

**Table 1:** Baseline characteristics of patients according to tertiles of the lowest nocturnal systolic blood pressure.

Characteristics	Overall (n = 356)	The lowest nocturnal systolic blood pressure, mmHg			*P* value
		≤ 102 (n = 118)	102–120 (n = 117)	≥ 120 (n = 121)	
**Demographic data**					
Age, years	66 [63, 72]	66 [63, 70]	66 [63, 72]	67 [63, 73]	0.154
Male, n (%)	237 (66.6)	69 (58.5)	78 (66.7)	90 (74.4)	**0.034**
BMI, kg/m^2^	25.1 [22.9, 27.3]	24.9 [22.5, 27.0]	25.2 [23.1, 27.7]	25.2 [23.1, 27.2]	0.327
Current smoker, n (%)	52 (14.6)	15 (12.7)	18 (15.4)	19 (15.7)	0.774
Hypertension, n (%)	263 (73.9)	71 (60.2)	89 (76.1)	103 (85.1)	** < 0.01**
Diabetes mellitus, n (%)	116 (32.6)	27 (22.9)	35 (29.9)	54 (44.6)	** < 0.01**
**CKD stages**					**0.026**
Stage 1, n (%)	58 (16.3)	25 (21.2)	17 (14.5)	16 (13.2)	
Stage 2, n (%)	79 (22.2)	25 (21.2)	26 (22.2)	28 (23.1)	
Stage 3, n (%)	132 (37.1)	46 (39.0)	45 (38.5)	41 (33.9)	
Stage 4–5, n (%)	87 (24.4)	22 (18.6)	29 (24.8)	36 (29.8)	
Kidney biopsy, n (%)	164 (46.1)	55 (46.6)	55 (47.0)	54 (44.6)	**0.925**
**Etiology of CKD**					**0.391**
CKD with uncertain reason, n (%)	139 (39.0)	54 (45.8)	45 (38.5)	40 (33.1)	
Membranous glomerulonephritis, n (%)	72 (20.2)	22 (18.6)	26 (22.2)	24 (19.8)	
Diabetic nephropathy, n (%)	37 (10.4)	6 (5.1)	8 (6.8)	23 (19.0)	
Hypertensive nephropathy, n (%)	24 (6.7)	8 (6.8)	6 (5.1)	10 (8.3)	
IgA nephropathy, n (%)	31 (8.7)	11 (9.3)	9 (7.7)	11 (9.1)	
Others, n (%)	53 (14.9)	17 (14.4)	23 (19.7)	13 (10.7)	
**Laboratory data**					
eGFR, ml/min per 1.73 m^2^	49.2 [30.2, 77.6]	57.0 [35.4, 86.3]	48.2 [29.9, 78.4]	45.3 [27.0, 68.6]	0.103
24 h proteinuria, g/day	1.8 [0.5, 3.9]	1.0 [0.3, 2.6]	1.6 [0.5, 3.2]	2.9 [1.2, 5.2]	** < 0.01**
Hemoglobin, g/l	122.0 ± 21.0	124.9 ± 21.4	121.8 ± 21.2	118.2 ± 19.8	**0.049**
Uric acid, μmol/l	402.1 ± 113.0	392.4 ± 112.6	421.7 ± 113.0	392.6 ± 111.8	0.073
Total cholesterol, mmol/l	5.0 [4.2, 6.2]	5.1 [4.1, 6.0]	4.9 [4.2, 5.9]	5.1 [4.3, 6.5]	0.171
TG, mmol/l	1.7 [1.2, 2.5]	1.7 [1.1, 2.6]	1.6 [1.1, 2.6]	1.7 [1.3, 2.3]	0.923
HDL, mmol/l	1.2 [1.0, 1.4]	1.1 [1.0, 1.4]	1.2 [1.0, 1.5]	1.2 [1.0, 1.4]	0.544
LDL, mmol/l	3.1 [2.4, 3.9]	3.1 [2.5, 3.8]	2.8 [2.2, 3.9]	3.3 [2.4, 4.3]	0.057
BUN, mmol/l	8.3 [6.4, 11.9]	7.8 [6.4, 11.4]	8.2 [6.3, 11.6]	8.9 [6.7, 12.7]	0.103
**Antihypertensive agents use**					
ACE inhibitors or ARB, n (%)	185 (52.0)	61 (51.7)	65 (55.6)	59 (51.2)	0.575
Diuretics, n (%)	38 (10.7)	9 (7.6)	15 (12.8)	14 (11.6)	0.403
Calcium channel blockers, n (%)	208 (58.4)	48 (40.7)	68 (58.1)	92 (76.0)	** < 0.01**
β-Blockers, n (%)	55 (15.4)	16 (13.6)	16 (13.7)	23 (19.0)	0.411
**ABPM parameters**					
24 h HR, /min	73 [67, 78]	74 [67, 79]	74 [67, 79]	71 [65, 77]	0.253
MAP, mmHg	92 [85, 100]	86 [79, 84]	91 [81, 96]	99 [94, 104]	** < 0.01**
24 h SBP, mmHg	130 [120, 141]	118 [109, 125]	127 [123, 132]	143 [136, 154]	** < 0.01**
24 h DBP, mmHg	74 [68, 79]	70 [63, 74]	73 [68, 78]	79 [74, 83]	** < 0.01**
Daytime SBP, mmHg	131 [120, 142]	119 [112, 130]	129 [122, 135]	143 [136, 154]	** < 0.01**
Daytime DBP, mmHg	75 [69, 80]	70 [63, 74]	73 [68, 78]	79 [74, 83]	** < 0.01**
Nighttime SBP, mmHg	127 [116, 142]	112 [102, 120]	124 [120, 130]	143 [136, 155]	** < 0.01**
Nighttime DBP, mmHg	71 [64, 77]	64 [54, 69]	71 [66, 75]	76 [72, 83]	** < 0.01**
Morning SBP, mmHg	134 [123,146]	121 [111, 130]	133 [125, 140]	148 [136, 159]	** < 0.01**
Office systolic blood pressure	133 [121, 147]	124 [112, 136]	133 [123, 145]	140 [130, 157]	** < 0.01**
Office diastolic blood pressure	79 [72, 85]	77 [71, 83]	79 [72, 85]	80 [74, 87]	**0.010**

Mean ± (SD) for normal distribution variables, median (interquartile range) for non-normal distribution variables, number (%) for category variables.

*eGFR* estimated glomerular filtration rate, *BMI* body mass index, *TG* triglyceride, *HDL* high-density lipoprotein, *LDL* low-density lipoprotein, *BUN* blood urea nitrogen, *ACE* angiotensin-converting-enzyme, *ARB* angiotensin receptor blockers, *CKD* chronic kidney disease, *24 h HR* 24 h heart rate, *SBP* systolic blood pressure, *DBP* diastolic blood pressure, *MAP* mean artery pressure.

### Association between the lowest nocturnal systolic blood pressure and heavy proteinuria in elderly CKD patients

**Figure 1:**
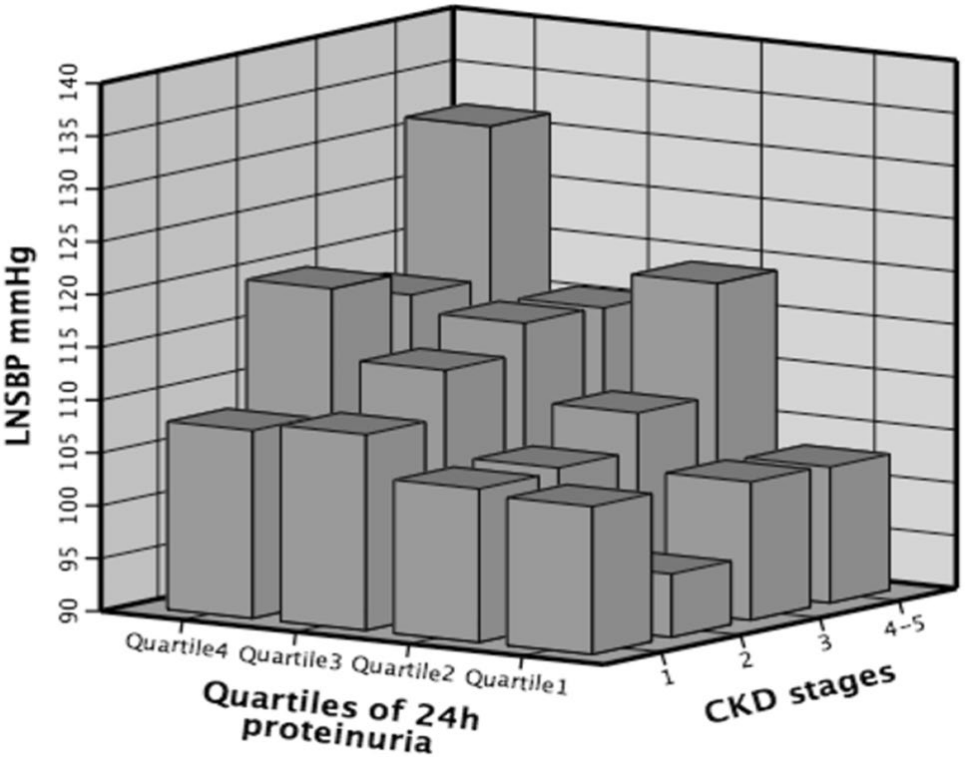
Influence of CKD stage and proteinuria on the lowest nocturnal systolic blood pressure (LNSBP). Quartile 4 represented superior proteinuria, and quartile 1 represented inferior proteinuria. Quartile 1: 24-h proteinuria < 0.5 g; Quartile 2: 0.5 g ≤ 24 h proteinuria < 1.8 g; Quartile 3: 1.8 g ≤ 24 h proteinuria < 4.0 g; Quartile 4: 24 h proteinuria ≥ 4.0 g.

We conducted logistic regression analysis to evaluate the relationship between LNSBP and heavy proteinuria (Table 2). Higher LNSBP, which was analyzed both as a continuous and categorical variable, was associated with higher proteinuria in all models. Every 10-mmHg increment in LNSBP (P < 0.001) increased the opportunity for heavy proteinuria by 24% (95% CI 1.10–1.39) after adjusting for background factors. Figure 1 showed the influence of CKD stage and proteinuria on the LNSBP. Patients with the heaviest proteinuria and the highest CKD stage had the highest LNSBP which was greater than 130 mmHg.

**Table 2:** The results of the logistic regression model to explain LNSBP related to heavy proteinuria.

Heavy proteinuria	Model 1			Model 2		Model 3	
	OR (95% CI)	*P* value		OR (95% CI)	*P* value	OR (95% CI)	*P* value
**Continuous**							
LNSBP (per 10 mmHg)	1.25 (1.11, 1.39)		** < 0.001**	1.24 (1.10, 1.39)	** < 0.001**	1.24 (1.10, 1.39)	** < 0.001**
**Tertiles (mmHg)**							
≤ 102	Reference			Reference		Reference	
102–120	1.25 (0.66, 2.36)	0.497		1.22 (0.64, 2.33)	0.547	1.27 (0.66, 2.43)	0.482
≥ 120	2.77 (1.54, 5.00)	**0.001**		2.81 (1.53, 5.15)	**0.001**	2.92 (1.55, 5.50)	**0.001**

Model 1: Univariable model; Model 2: Adjusted for age and sex; Model 3: Model 2 + current smoking, *eGFR* history of hypertension or diabetes, and etiology of CKD.

*LNSBP* the lowest nocturnal systolic blood pressure.

### Associations of the lowest nocturnal systolic blood pressure with outcomes

**Figure 2:**
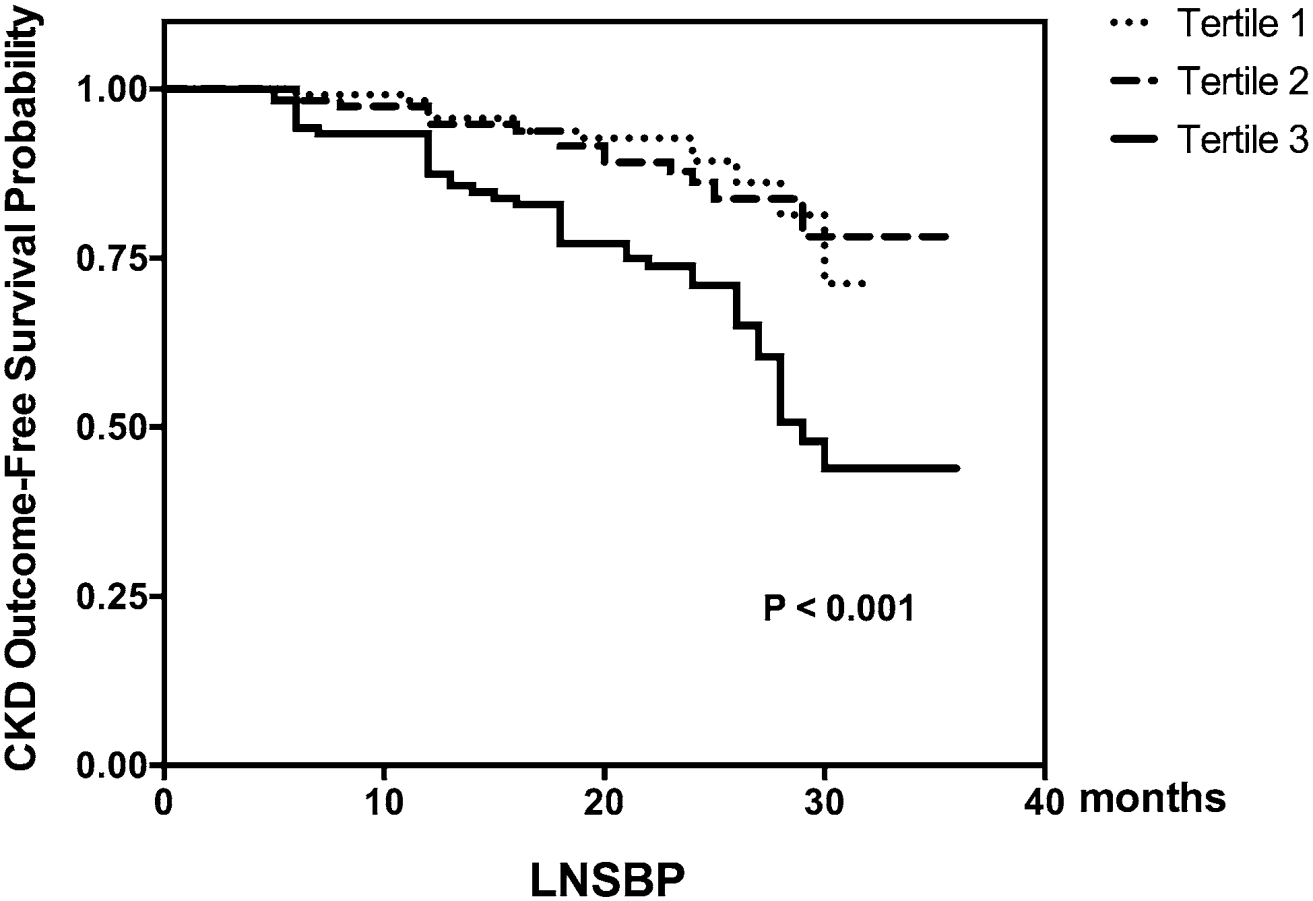
Kaplan–Meier analysis of composite outcome-free survival probability estimated by the lowest nocturnal systolic blood pressure. Kaplan–Meier curves stratified tertiles of the LNSBP. Time to events differed significantly between groups (log-rank P < 0.001).

During the median follow-up of 23 months, 70 patients (19.7%) had a composite outcome. Of those, 25 initiated dialysis, 25 had 40% eGFR loss from baseline, and 20 died. In Kaplan–Meier (KM) analysis (Fig. 2), the time functions for LNSBP predicting CKD composite outcome-free survival probability were significantly different (P < 0.01). The patients with the highest LNSBP tertile were more likely to have poor prognoses during follow-up. Cox proportional hazards models consistently showed that LNSBP was independently associated with kidney disease outcomes, including CKD progression and death, after adjusting for age, sex, current smoking, medical history of hypertension and diabetes, eGFR, 24-h proteinuria, and etiology of CKD, as illustrated in Table 3. A 10-mmHg increase in the LNSBP values conferred an 18% (95% CI 1.06–1.32; P = 0.002) increase in the risk of developing CKD composite events and a 24% (95% CI 1.08–1.41; P = 0.002) increase in CKD progression (initiation of replacement treatment therapy and 40% eGFR decline) when death was considered as censored data.

**Table 3:** Hazard ratio (HRs) (95% CI) for clinical outcomes (ESRD, 40% eGFR decline or death) per 10 mmHg increase in LNSBP in elderly CKD patients.

ABPM	CKD progression or death			CKD progression (death as censored data)		
	HR	95%CI	*P* value	HR	95% CI	*P* value
**Continuous**						
LNSBP (per 10 mmHg)	1.18	(1.06, 1.32)	**0.002**	1.24	(1.08, 1.41)	**0.002**
**Tertiles (mmHg)**						
≤ 102	**Reference**			**Reference**		
102–120	1.07	(0.50, 2.29)	**0.867**	1.10	(0.44, 2.72)	**0.843**
≥ 120	2.36	(1.23, 4.54)	**0.010**	2.74	(1.23, 6.09)	**0.013**

Model adjusted for age, gender, smoking status, medical history of hypertension and diabetes, estimated GFR, 24 h proteinuria, etiology of CKD.

*LNSBP* the lowest nocturnal systolic blood pressure.

## Discussion

To the best of our knowledge, this is the first study emphasizing the association between LNSBP and heavy proteinuria and the risk of LNSBP for kidney disease outcomes.

A well-established association between ABPM and microalbuminuria has been reported in some previous studies^16–18^. A cross-sectional study described that higher nighttime blood pressure indicated higher albuminuria in diabetes and CKD patients^19^. However, the authors defined albuminuria (albumin creatinine rate, ACR > 300 mg/g) as a very high level of proteinuria instead of using a 24-h urine test, which is a method used to assess the quantity of all kinds of urine protein. In addition, we explored the lowest systolic blood pressure as either a continuous variable or a categorical variable to predict heavy proteinuria and obtained a positive result. Several studies reported that ABPM variables, particularly nighttime blood pressure, were strongly associated with the progression of microalbuminuria in individuals at high risk of CKD^17,20,21^, and our data added evidence that nighttime blood pressure was also associated with heavy proteinuria.

Hypertensive nephropathy is the second leading cause of CKD in the majority of countries^22^. Recently, a new viewpoint contrasting with previous thinking that elevated proteinuria is secondary to the development of high BP has been justified gradually^23–26^. Brantsma et al.^21^ conducted a longitudinal study with 4635 normotensive individuals at baseline to investigate the relationship between urinary albumin excretion and the new onset of hypertension. They found that participants with higher urinary albumin excretion at baseline were more likely to develop hypertension (OR 1.18; per 1-unit In (UAE) change; 95% CI 1.03–1.36) after a mean 4.2 year of follow-up. In 2018, Haas et al*.*^26^ examined genetically bidirectional associations of proteinuria with blood pressure and suggested that elevated proteinuria and blood pressure could affect each other, which meant that increased proteinuria would lead to higher blood pressure and vice versa. It is reasonable for us to understand that sustained hypertension can lead to proteinuria. However, how can hypertension be explained by proteinuria? The mechanisms accounting for this interaction remain unclear. However, there are several speculations here. First, proteinuria was considered the result of kidney damage and seemed to be a major risk factor and pathological stimulus of renal inflammation-causing impairment in sodium homeostasis and overload, which led to hypertension^27,28^. Second, proteinuria might arise due to generalized endothelial dysfunction, which may also cause hypertension^29,30^. Third, urinary serine proteases aberrantly filtered into tubules, such as plasmin, activate the epithelial sodium channel in the distal tubule, with which sodium retention and volume overload may contribute to hypertension^24,31^. Combining with previous studies, we indeed think there is an underlying link between night blood pressure and heavy proteinuria, and a higher nighttime blood pressure level can be strongly associated with heavier proteinuria or the converse association.

The LNSBP is mostly used in calculating morning surges^32,33^. However, studies illustrating the physiological meaning of LNSBP are rare. Cesare Cuspidi et al.^34^ reported that the LNSBP was predictive of new-onset left ventricular mass during a 10-year follow-up period. Combining our data with published literature, on the one hand, in terms of physical and mental activity as well as body position, LNSBP represents the minimal BP needed for adequate organ perfusion and physiological activity. On the other hand, in terms of arterial structure and elasticity, LNSBP reflects the degree of vascular stiffness. A higher LNSBP indicates a greater chance of target organ damage. In our study, one-third of elderly CKD patients had a nocturnal systolic blood pressure of greater than 120 mmHg, which far exceeds 90/60 mmHg, the lowest organ perfusion requirement in a healthy population. We confirmed that in elderly CKD patients, high LNSBP may be attributed to high basal systolic blood pressure or arterial stiffness free from interference of diurnal activities, hormone changes, sympathetic nervous tone, and the renin–angiotensin system. However, further research on relative explorations and comparison is warranted.

Our study suggested an increased risk of poor prognosis in elderly CKD patients with higher LNSBP independent of conventional CKD progression risk factors. Nighttime BP has been explored in many studies, and most of them have concluded that nighttime BP is superior in indicating ESRD or death than daytime BP and office blood pressure in CKD or non-CKD patients^35–37^. Although the pathophysiological mechanism of the association between nocturnal blood pressure and worsening renal prognosis has not been confirmed, our study demonstrated that higher LNSBP was associated with heavier proteinuria, which can accelerate renal function loss. In addition, LNSBP was independently associated with prognosis, indicating that higher organ blood perfusion or the degree of arterial stiffness can affect the hemodynamics of the kidney and lead to renal function loss.

Given that heavy proteinuria and prognosis are the priorities for nephrologists, it is reasonable to measure the lowest nighttime blood pressure by ABPM devices. Proper administration of anti-night hypertensive treatment may help to control proteinuria and slow the progression of CKD in the elderly population.

Several limitations should be mentioned. First, only elderly patients were enrolled in this study, and population representation was relatively limited. Second, 70 outcomes, including only 20 all-cause deaths, occurred during follow-up, which prevented us from detecting an association between LNSBP and all-cause death. Patient number did not provide sufficient power to evaluate the antihypertensive drug effects on LNSBP. Third, some of the day and night durations were defined by wide fixed-clock intervals (6:00 AM–10:00 PM for the day and 10:00 PM–6:00 AM for the night). Some patients may be asleep, whereas others may be awake, representing a source of data inaccuracy. Some sleep durations were self-reported, which was probably misreported by patients, resulting in potential errors in some ABPM values. Fourth, 24-h urine collection and ABPM were both performed only once at baseline, and changes in blood pressure and proteinuria during follow-up may influence the results. Finally, we did not analyze the association between LNSBP and proteinuria during follow-up due to the susceptibility of proteinuria to environmental factors, drugs, physical activities, and diets. Thus, the causal relationship between LNSBP and proteinuria has not been studied. In conclusion, higher nocturnal systolic BP was independently associated with an increased risk for heavy proteinuria and poor outcomes. Concentrating on the lowest nocturnal systolic blood pressure could be valuable in guiding antihypertensive treatment to control heavy proteinuria and improve renal prognosis in elderly CKD patients.

## Methods

### Study design and participants

This study was a multicenter, observational cohort study that was launched in February 2017 to investigate elderly Chinese CKD patients (greater than 60 years of age) recruited from 19 clinical centers in China. All the participants conformed to the following inclusion criteria: aged ≥ 60 years old, received a diagnosis of CKD^38^ and provided signed informed consent. We excluded participants if they (1) had received dialysis or renal transplantation; (2) were diagnosed with acute kidney injury; (3) had active malignancy within 24 months prior to screening or metastatic cancer; (4) had a life expectancy less than 6 months; (5) had severe heart failure (New York Heart Association function class III or IV); (6) had HIV infection; (7) had isolated hematuria; and (8) were unable to communicate with examiners, unable to complete the study procedure even if assisted or otherwise unable to comply with the study protocol. A total of 356 predialysis patients with ABPM records and basic information were enrolled until February 2019. The CKD Epidemiology Collaboration (CKD-EPI) creatinine equation^39^ was adopted to calculate the estimated glomerular filtration rate (eGFR). This study was approved by the Ethics Committee of the Chinese PLA General Hospital (No. S2016-100-02). All participants signed informed consent before they joined this study.

### 24-h proteinuria and 24-h ABPM

Our patients started 24-h urine collections after their first voiding in the morning and to collect all urine continuously for 24 h, including the last void at the end of the 24-h period, or timely collected 24-h urine from 7:00 to 7:00 AM of the second day. Twenty-four-hour urine total protein was assayed by using the pyrogallol red method (Beijing Strong Biotechnologies, China). Urine volume less than 500 ml was excluded.

ABPM was performed by skilled physicians with validated devices (H-1184 Budapest, Hungary) on a routine working day after the patients provided informed consent. The monitors recorded blood pressure at 15-min intervals in the daytime and 30-min intervals in the nighttime for a 24-h period. Different sized cuffs were used based on the arm circumference of the patients. Patients with monitors were suggested to maintain their usual activities. Valid measurements were those fulfilling ≥ 80% continuous recordings. Day and night durations were defined by wide fixed-clock intervals (6:00 AM–10:00 PM for the day and 10:00 PM–6:00 AM for the night) or according to self-reports of patients. The lowest nocturnal systolic blood pressure (LNSBP) was defined as the mean of the three nighttime SBP readings, the lowest reading, and the readings immediately before and after, centered on the time of the lowest nighttime values. Morning SBP (MSBP) was defined as the average of SBP values during the first 2 h after wake-up or the mean value obtained between 06:00 AM and 08:00 AM.

### Other variables and definitions

Apart from 24-h proteinuria and ABPM, we also collected data, including age, sex, smoking status, body mass index (BMI), medical history of hypertension and diabetes, serum creatinine, eGFR, total cholesterol (TC), triglyceride (TG), high-density lipoprotein cholesterol (HDL), low-density lipoprotein cholesterol (LDL), blood urea nitrogen (BUN), uric acid, hemoglobin, and antihypertensive medication history.

CKD stages were consistent with the Kidney Disease Improving Global Outcomes (KDIGO) 2012 guidelines^38^. Specific causes of CKD were mainly dependent on pathologic diagnosis when the biopsy result was available or otherwise dependent on the clinical diagnosis made by nephrologists. All enrolled patients were categorized into the following subgroups: CKD with uncertain reason, membranous nephropathy, IgA nephropathy, diabetic nephropathy, hypertensive nephropathy, and others. The patients with other specific renal biopsy diagnoses were categorized as “others”. Hypertension was defined as office blood pressure (BP) ≥ 140/90 mmHg, self-reported hypertension history, or current treatment with antihypertension drugs. Diabetes mellitus was defined as a fasting glucose level ≥ 7.0 mmol/l, glycated hemoglobin ≥ 6.5%, self-reported diabetic history, or current treatment with antidiabetic drugs.

### Outcomes

CKD progression was defined as ESRD (the initiation of replacement treatment therapy) or 40% eGFR decline, which ever occurred first. We selected a 40% eGFR decline over follow-up because this outcome was clinically significant and proposed by the National Kidney Foundation and the US Food and Drug Administration as a broadly accepted measure for kidney function decline^40^. CKD progression and all-cause death were composite outcomes. CKD progression and all-cause death were ascertained through contact with patients or reports from next of kin, review of medical records and death certifications. Individuals should visit the clinics every 6 months. The allowance of the early and delayed visits would be 1 month from the scheduled visit date. Patients who were not able to show up in hospitals on time were followed up by telephone call or online contact until ESRD, death, withdrawal from the study, loss to follow-up, or January 2020. Greater than 90% of the patients kept in touch during this period. All methods were performed in accordance with the relevant guidelines and regulations.

### Statistical analysis

We summarized baseline participant characteristics across tertiles of baseline the lowest nocturnal systolic blood pressure with mean ± (SD) for normal distribution variables, median (interquartile range) for nonnormal distribution variables, number (%) for category variables. We categorized proteinuria as a binary variable [nonheavy proteinuria (< 3.5 g/24 h) part and heavy proteinuria (≥ 3.5 g/24 h)] because the data did not meet the requirement for normal distribution of linear regression. Multiple logistic regression analysis was used to investigate the association between ABPM parameters and heavy proteinuria. Kaplan–Meier survival curves were used to estimate the proportion of participants in different tertiles of LNSBP surviving without a composite outcome for specified durations. Cox proportional hazards regression was used to calculate hazard ratios (HRs) and 95% confidence intervals (95% CIs) for time to renal outcomes with adjustment for traditional risk factors, including age, sex, smoking status, medical history of hypertension and diabetes, eGFR, 24-h proteinuria, and etiology of CKD in either death as an outcome or censored data models. The assumption of proportionality was tested using Schoenfeld residuals. No substantial deviations from proportionality were observed. Statistical significance was set at the level of P = 0.05. Analyses were performed with IBM SPSS 25.0 software (IBM Corp., Armonk, NY, USA).
